# Analysis of Functional Differences between Hepatitis C Virus NS5A of Genotypes 1–7 in Infectious Cell Culture Systems

**DOI:** 10.1371/journal.ppat.1002696

**Published:** 2012-05-24

**Authors:** Troels K. H. Scheel, Jannick Prentoe, Thomas H. R. Carlsen, Lotte S. Mikkelsen, Judith M. Gottwein, Jens Bukh

**Affiliations:** Copenhagen Hepatitis C Program (CO-HEP), Department of Infectious Diseases and Clinical Research Centre, Copenhagen University Hospital, Hvidovre and Department of International Health, Immunology and Microbiology, Faculty of Health Sciences, University of Copenhagen, Copenhagen, Denmark; University of California, San Diego, United States of America

## Abstract

Hepatitis C virus (HCV) is an important cause of chronic liver disease. Several highly diverse HCV genotypes exist with potential key functional differences. The HCV NS5A protein was associated with response to interferon (IFN)-α based therapy, and is a primary target of currently developed directly-acting antiviral compounds. NS5A is important for replication and virus production, but has not been studied for most HCV genotypes. We studied the function of NS5A using infectious NS5A genotype 1–7 cell culture systems, and through reverse genetics demonstrated a universal importance of the amphipathic alpha-helix, domain I and II and the low-complexity sequence (LCS) I for HCV replication; the replicon-enhancing LCSI mutation S225P attenuated all genotypes. Mutation of conserved prolines in LCSII led to minor reductions in virus production for the JFH1(genotype 2a) NS5A recombinant, but had greater effects on other isolates; replication was highly attenuated for ED43(4a) and QC69(7a) recombinants. Deletion of the conserved residues 414-428 in domain III reduced virus production for most recombinants but not JFH1(2a). Reduced virus production was linked to attenuated replication in all cases, but ED43(4a) and SA13(5a) also displayed impaired particle assembly. Compared to the original H77C(1a) NS5A recombinant, the changes in LCSII and domain III reduced the amounts of NS5A present. For H77C(1a) and TN(1a) NS5A recombinants, we observed a genetic linkage between NS5A and p7, since introduced changes in NS5A led to changes in p7 and vice versa. Finally, NS5A function depended on genotype-specific residues in domain I, as changing genotype 2a-specific residues to genotype 1a sequence and vice versa led to highly attenuated mutants. In conclusion, this study identified NS5A genetic elements essential for all major HCV genotypes in infectious cell culture systems. Genotype- or isolate- specific NS5A functional differences were identified, which will be important for understanding of HCV NS5A function and therapeutic targeting.

## Author Summary

Hepatitis C virus (HCV) is a major public health burden and leads to chronic liver disease, including liver cirrhosis and liver cancer. Understanding the biological functions of the virus is crucial to the development of a vaccine and to improve current therapy through development of directly-acting antiviral compounds. The NS5A protein is a promising antiviral target, but much remains to be understood about its role in the viral life cycle. Great diversity among the seven major HCV genotypes poses challenges for broadly active inhibitors. Here we used infectious cell culture systems for NS5A of the seven major HCV genotypes, and demonstrated that all genotypes depended on the NS5A amphipathic alpha-helix, domain I, low-complexity sequence (LCS) I and domain II for viral replication. Interestingly, effects on replication and virus production by changes in LCSII and domain III varied greatly among NS5A isolates. Furthermore, we found that genotype 2 had evolved genotype-specific residues in domain I of importance for viral function. Thus, the highly diverse sequence of the NS5A protein reflected functional differences between HCV genotypes and isolates. Such differences will be important to consider in understanding HCV biology and for future development of antiviral compounds.

## Introduction

Hepatitis C virus (HCV) chronically infects 130–170 million people and leads to increased risk of severe liver disease. HCV belongs to the *Flaviviridae* family and has a positive-strand RNA genome containing one long open reading frame (ORF). The ORF encodes a polyprotein that is co- and post-translationally cleaved into structural proteins (Core, E1, E2), p7 and nonstructural (NS) proteins NS2, NS3, NS4A, NS4B, NS5A and NS5B. Significant diversity is found among HCV isolates, which in phylogenetic analysis cluster into seven major genotypes and numerous subtypes [1], [2]. Genotypes, subtypes and isolates/strains differ at around 30%, 20% and 2–10%, respectively, at the nucleotide and amino acid level. A higher variability is found in certain genome regions. Among different genotypes, the NS5A protein sequence varies up to 50% in composition and by more than 20 residues in length. Important differences between HCV genotypes were identified in biology [3] and in sensitivity to neutralizing antibodies [4]–[7]. The HCV genotype is an important determinant for response to the current interferon (IFN)-α based treatment regimens; sustained virological response is achieved for 80–90% of genotype 2 and 3 and for around 50% of genotype 1 and 4 infected patients [8]. Several HCV genes, including E2, NS3 and NS5A, were suggested to influence the response to IFN [9]. Directly-acting antiviral compounds are currently being developed with the NS3 protease, the NS5B polymerase and NS5A as primary targets [10]. Genotype- and isolate-specific responses to treatment with directly-acting antivirals have been reported in vitro [11], [12] and in clinical trials [13], [14].

The NS5A phosphoprotein is a component of the viral replication complex [15] and consists of three domains separated by low-complexity sequences (LCS) [16]. It is anchored to intracellular membranes through the N-terminal amphipathic alpha-helix [17], [18]. A crystal structure was solved for domain I [19], [20], which contains a zinc-binding motif [16] and a highly basic channel with RNA binding capacity [21]. The amphipathic alpha-helix, domain I and II are essential for the genotype 1b replicon system [16], [22], [23]. In LCSI and domain II, covering parts of the binding site for the IFN-induced dsRNA-dependent protein kinase R (PKR) [24], an IFN-sensitivity determining region (ISDR) was described for genotype 1b [25], although, its existence is controversial [26]. Several studies on genotype 2a suggest a primary role for domain III in production of infectious particles [27]–[29]. For most genotypes, however, the role of NS5A in the viral life cycle has not been studied.

Until development of the genotype 2a JFH1 cell culture system [30], and the more efficient J6/JFH1 system with the Core-NS2 region from another 2a isolate [31], studies on HCV NS5A relied on the genotype 1b and 2a replicon systems, recapitulating only parts of the viral life cycle [9]. J6/JFH1-based infectious cell culture systems for NS5A genotypes 1–7 [12] allowed us to study the function of the highly variable NS5A protein for all major HCV genotypes in context of the complete viral life cycle. We analyzed individual NS5A domains for their influence on steps of the viral life cycle and found genotype- and isolate-specific effects of introduced mutations and modifications.

## Results

### All seven major HCV genotypes depend on the NS5A amphipathic alpha-helix, domain I, LCSI and domain II for viral replication

**Figure 1:**
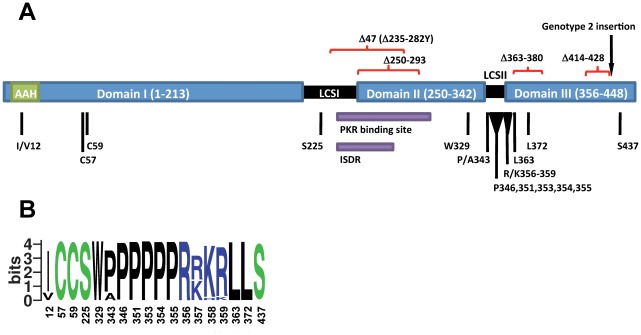
HCV NS5A residues and regions analyzed in reverse genetic studies. Numbering is according to the H77 reference NS5A sequence (GenBank accession number AF009606). (A) Schematic drawing of the NS5A protein. The putative domains I–III (blue), are linked by LCSI and II (black). The amphipathic alpha-helix (AAH) in domain I is predicted to cover residues 5–25 (green) [18]. A putative IFN-sensitivity determining region (ISDR) covering residues 237–276 [25] and a PKR binding region covering residues 237–302 [24] (purple) extend from the LCSI into domain II. Positions mutated in reverse genetic studies are indicated in black and deletions are indicated in red. An NS5A amino acid alignment of isolates studied is shown in Figure S1. (B) Variation at positions mutated in reverse genetic studies among isolates in the most recent pre-made NS5A web-alignment consisting of 673 entries from the Los Alamos HCV sequence database visualized as logo plot [63]. The information content at every position is given in bits. Amino acids are colored by properties: polar (green), basic (blue), acidic (red) and hydrophobic (black). In addition to the residues shown, a few serines were found at residues 346 and 354, while a few asparagines were found at residue 437. Residues observed in less than two sequences were not included.

To determine the importance of the individual NS5A domains for the major HCV genotypes in context of the complete viral life-cycle, we introduced selected mutations (Figure 1A) into J6/JFH1-based NS5A genotype 1–7 recombinants, which we previously demonstrated were infectious and efficient in vitro (Materials & Methods) [12]. In previous studies, it was found that the Con-1(genotype 1b) replicon system was inhibited by the I12E mutation shown to disrupt the hydrophobic face of the amphipathic alpha-helix [22], by C57G and C59G mutations interfering with zinc-ion binding of domain I [16], and by mutating the conserved W329 in domain II [23] (Numbering throughout is according to individual H77 reference proteins, GenBank accession number AF009606). The S225P mutation in LCSI was shown not to be permissible in vivo when introduced into the Con-1(1b) full-length infectious clone [32], even though it had an enhancing effect on the Con-1(1b) replicon system [33], [34]. These residues were all highly conserved among HCV patient isolates (Figure 1B). To analyze the effect of these mutations in cell culture, RNA transcripts of more than 30 mutants of the H77C(1a), J4(1b), JFH1(2a), S52(3a), ED43(4a), SA13(5a), HK6a(6a) and QC69(7a) NS5A recombinants were transfected into Huh7.5 cells in parallel with the positive control J6/JFH1 (for consistency denoted JFH1(2a) according to the NS5A isolate) [31]. While around 30% of cells were HCV positive one day after JFH1(2a) transfection, the I/V12E, C57G/C59G, S225P and W329A genotype 1–7 mutants were all highly attenuated, as no or very few HCV positive cells were observed in immunostainings one and three days post-transfection.

Since reversion was observed in vivo for the Con-1(1b) S225P mutant coinciding with detection of high viral titers [32], we followed the J4(1b) S225P mutant until it, after two weeks, infected most cells. In virus recovered after passage to naïve cells, S225P had reverted. Thus, for this mutant the findings in the infectious cell culture system were in accordance with findings in vivo but not with findings in the replicon system [33], [34]. To determine whether another mutant with a replicon-enhancing alteration was also attenuated we changed the J4(1b) NS5A recombinant to encode the Δ47 deletion, which replaces residues 235–282 in NS5A LCSI/domain II by a single tyrosine (Figure 1A) [33]; this mutant was followed for three weeks without detection of HCV positive cells. Thus, the positive effect of NS5A replicon-enhancing mutations apparently led to the opposite effect in the infectious cell culture system.

We further investigated whether the phenotype observed for the highly attenuated NS5A mutants corresponded to that of mutants of other HCV nonstructural proteins previously shown to abrogate HCV RNA replication. No HCV positive cells were observed one day after transfection of JFH1(2a) mutants of the NS3 protease active site (NS3pro^−^, S139A [35]), the NS3 helicase active site (NS3hel, D290A [35]), the NS4A transmembrane segment (G21V [36]), the NS4B C-terminal end (W252S [37]), the NS5B polymerase active site (GND-mutant, D318N [38]), for a JFH1(2a) mutant with a stop codon in the NS3 N-terminus (Y6[stop]), or for the JFH1(2a) NS5A domain I mutants C57G, C59G or C57G/C59G included for comparison. However, infection emerged subsequently for the NS3_hel_, NS4B_W252S_, NS5A_C59G_, NS5B_GND_ and NS3_Stop_ mutants when following cultures for more than two weeks. Reversion of the knockout mutations and the presence of silent marker mutations engineered to exclude contamination were confirmed by sequencing. We found that in a total of 13 experiments done with NS5B_GND_, 10 led to emerging infection. Viruses recovered from five of these cultures were subsequently sequenced, in all of which the attenuating mutation had reverted; the marker mutations were maintained. Thus, the phenotype of attenuated NS5A mutants corresponded to that of single mutations of other non-structural genes expected to be detrimental for viral replication. For these mutants, reversion was detected in most cases. We speculate that this was due to transfected RNA pools containing genomes that were reverted to wild-type as a result of the high error-rate of T7 polymerase driven in vitro RNA transcription.

### Isolate-specific effect of an N-terminal deletion in NS5A domain II on viability

**Figure 2:**
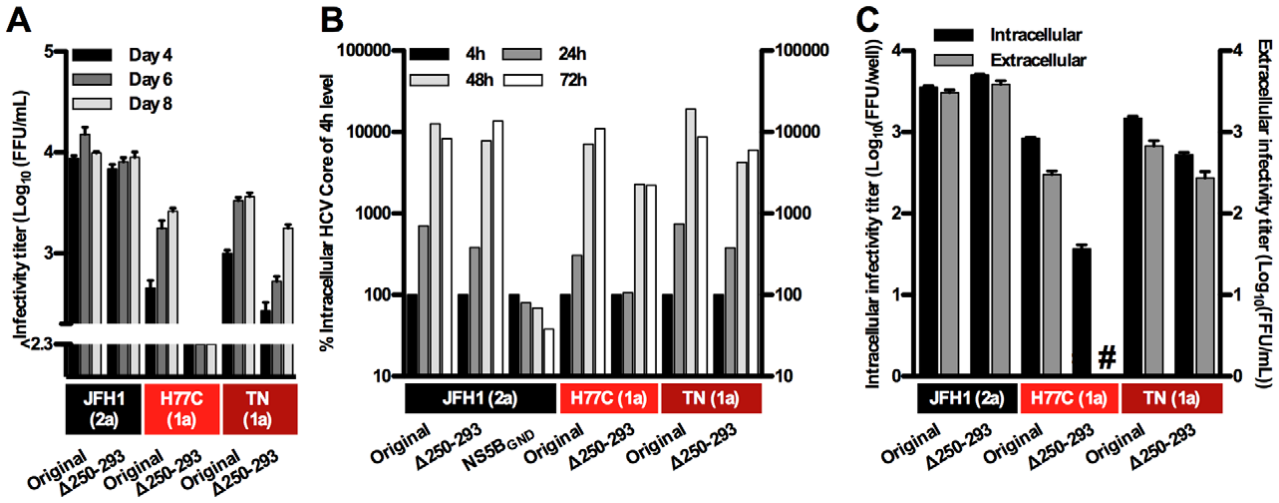
Infectivity titers and replication capacity of NS5A genotype 1a and 2a recombinants with N-terminal deletion in domain II. (**A**) Infectivity was measured in supernatants after transfection of Huh7.5 cells with RNA transcripts from H77C(1a), TN(1a) or JFH1(2a) with and without deletion of residues 250–293 (Δ250–293) (Figure S1). The lower limit of detection in the experiments shown was 10^2.3^ FFU/mL (indicated by the y-axis break). (**B**) As a measure for HCV RNA replication, S29 cells were transfected for 4 hours and intracellular HCV Core levels were measured after 4, 24, 48 and 72-hours. Values were normalized for transfection efficiency using 4-hour Core amounts of the respective recombinant. The JFH1(2a) NS5B_GND_ mutant was included as a replication negative control. (**C**) Intra- and extracellular infectivity titers 48 hours after transfection of S29 cells. Intracellular titers are given per well of 4×10^5^ transfected S29 cells. #: no FFU were detected. Colored boxes indicate the NS5A isolate and (genotype). Error bars indicate SEM of triplicate determinations.

The W329 in the C-terminal end of NS5A domain II was found to be of critical importance for replication of NS5A genotype 1–7 recombinants, while it was previously reported that a JFH1 NS5A mutant with a deletion of residues 246–308 covering a large N-terminal region of domain II (Δ2222–2280 mutant) apparently was fully viable [27]. We had similar findings for a J6/JFH1 virus without NS5A residues 250–293 [39], and therefore wanted to investigate whether deletion of the corresponding residues was generally permitted for NS5A isolates. Thus we constructed H77C(1a) and TN(1a) Δ250–293 mutants (Figure 1A and S1) and transfected RNA transcripts into Huh7.5 cells. Infectivity titers after transfection were reduced for these mutants, most significantly for H77C(1a); as previously observed, titers for the JFH1(2a) mutant were not decreased (Figure 2A). This corresponded to immunostainings with 20–30% HCV positive cells one day post-transfection, except for the H77C(1a) Δ250–293 mutant, for which only 5% were positive. All recombinants spread to the majority of cells within 8 days. Analysis of NS5A of recovered passaged viruses of JFH1(2a), H77C(1a) and TN(1a) Δ250–293 mutants confirmed the deletion. Amino acid changes D444G (D2416G) and C447R (C2419R) were identified in H77C(1a) and TN(1a) Δ250–293, respectively. No NS5A changes were observed for the JFH1(2a) mutant.

To investigate whether viral replication, assembly or release was affected for H77C(1a) and TN(1a) Δ250–293 mutants, we transfected CD81-deficient Huh7-derived S29 cells that are not susceptible to HCV infection [40]. HCV Core levels were used as a measure for RNA replication [41]; validation experiments confirmed the correlation between intracellular HCV RNA and Core levels (Figure S2). From separate cultures, assembly and release was evaluated by titration of intra- and extra-cellular infectivity. While replication and virus production was not affected for the JFH1(2a) mutant, minor decreases in replication levels and intracellular virus production was observed for TN(1a) (Figure 2B and C). The H77C(1a) mutant exhibited greater reductions, in particular for intra- and extra-cellular virus production that was reduced more than 10-fold. Thus, while the C-terminal region of domain II was critical for replication of all HCV genotypes, deletion of the N-terminal residues 250–293 led to a highly attenuated phenotype for H77C(1a), an intermediate phenotype for TN(1a) and no attenuation of JFH1(2a).

### Isolate-specific dependence on conserved prolines in NS5A LCSII for replication and virus production

**Figure 3:**
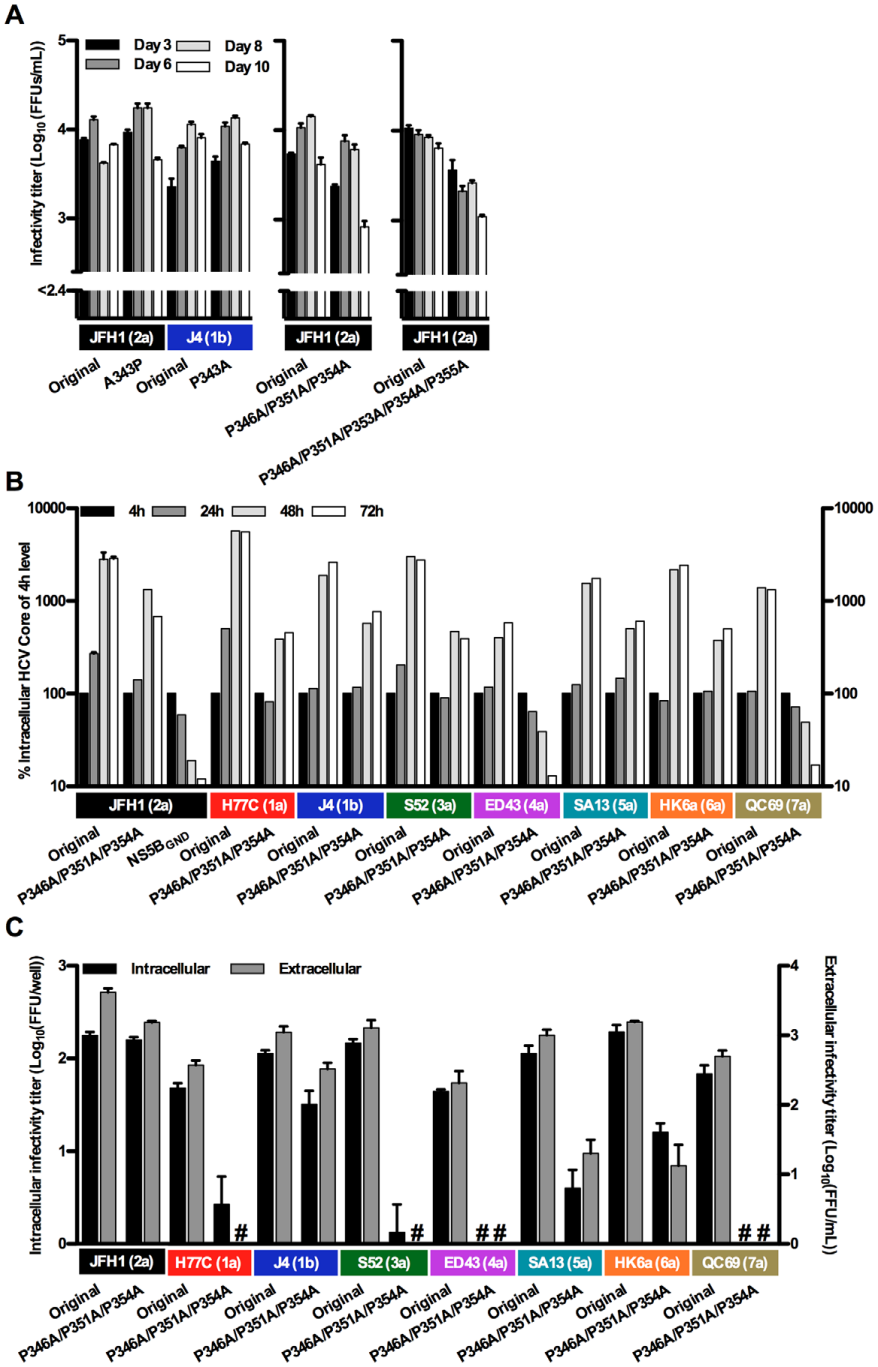
Infectivity titers and replication capacity of HCV NS5A LCSII mutants. (**A**) Infectivity titers in supernatant after transfection of Huh7.5 cells with original NS5A genotype recombinants or LCSII mutants. For the residue 343 mutants, J4 and JFH1 recombinants are shown as representative examples, since no significant effect on titers was observed for any of the NS5A genotype 1–7 recombinants. For comparison, JFH1(2a) was included in each experiment. The lower limit of detection in the experiments shown was 10^2.4^ FFU/mL (indicated by the y-axis break). (**B**) As a measure for HCV RNA replication, S29 cells were transfected for 4 hours with original NS5A genotype recombinants and P346A/P351A/P354A mutants, and intracellular HCV Core levels were measured after 4, 24, 48 and 72-hours. Values were normalized for transfection efficiency using 4-hour Core amounts of the respective recombinant. The JFH1(2a) NS5B_GND_ mutant was included as a replication negative control. (**C**) Intra- and extracellular infectivity titers 48 hours after transfection of S29 cells with original NS5A genotype recombinants and P346A/P351A/P354A mutants. Intracellular titers are given per well of 4×10^5^ transfected S29 cells. #: no FFU were detected. Colored boxes indicate the NS5A isolate and (genotype). Error bars indicate SEM of triplicate determinations, except for in (B) where JFH1(2a) was included in duplicates.

The NS5A LCSII region was shown to influence HCV replication and virus production [23], [42]. The Con-1(1b) replicon system was reported to be highly attenuated by the P343A LCSII mutation [23]. However, changes at this genotype-specific position (P343A for genotype 1 and 5, A343P for other genotypes, Figure S1) did not influence infectivity titers after transfection of Huh7.5 cells for any of the NS5A genotype 1–7 recombinants (Figure 3A and data not shown). No changes were identified in the complete ORF of the recovered JFH1(2a) A343P mutant, and residue 343 had not reverted for mutants of other NS5A isolates. This was in agreement with findings in a more recent Con-1(1b) replicon study [42].

In addition, we generated the JFH1(2a) P346A/P351A/P354A and P346A/P351A/P353A/P354A/P355A LCSII mutants, in which highly conserved prolines (Figure 1B and S1) in two putative SH3 interaction domains [42]–[44] were mutated. Infectivity titers after transfection of Huh7.5 cells were decreased <10-fold (Figure 3A), and the mutations did not revert after passage to naïve cells. Next, the P346A/P351A/P354A mutations were introduced into the NS5A genotype 1–7 recombinants, and tested for viability in Huh7.5 cells. While supernatant HCV infectivity titers were only slightly decreased for the J4(1b) mutant, reductions by 10-fold or more were observed for H77C(1a), S52(3a), ED43(4a), SA13(5a), HK6a(6a) and QC69(7a) mutants (data not shown). To analyze whether attenuation was caused by reduced replication capacities, we measured intracellular HCV Core after transfection of S29 cells. RNA replication of ED43(4a) and QC69(7a) P346A/P351A/P354A mutants was highly attenuated, with intracellular HCV Core levels similar to those observed for the NS5B_GND_ mutant (Figure 3B). These findings were in agreement with immunostainings, which on day 1 after transfection of Huh7.5 cells were HCV negative for the ED43(4a) and QC69(7a) mutants. For all other LCSII mutants, replication was decreased with up to 10-fold reductions in intracellular HCV Core levels 48 and 72 hours post-transfection. While intra- and extra-cellular infectivity titers from transfected S29 cells were only slightly decreased for the J4(1b) and JFH1(2a) mutants (Figure 3C), greater than 10-fold reductions were observed for the H77C(1a), S52(3a), SA13(5a) and HK6a(6a) mutants. Thus the P346A/P351A/P354A mutations had a profound impact on intracellular virus production for some NS5A isolates but not for others. No infectivity was observed for the ED43(4a) and QC69(7a) mutants, as expected from RNA replication assays. Thus, various NS5A isolates tolerated changes in LCSII to different extents. The ED43(4a) and QC69(7a) mutants were highly attenuated in viral replication compared to other isolates, while assembly of intracellular infectious viral particles was affected by LCSII mutations at an isolate-specific level.

### The importance of a conserved region in NS5A domain III for replication and virus production depends on the NS5A isolate

**Figure 4:**
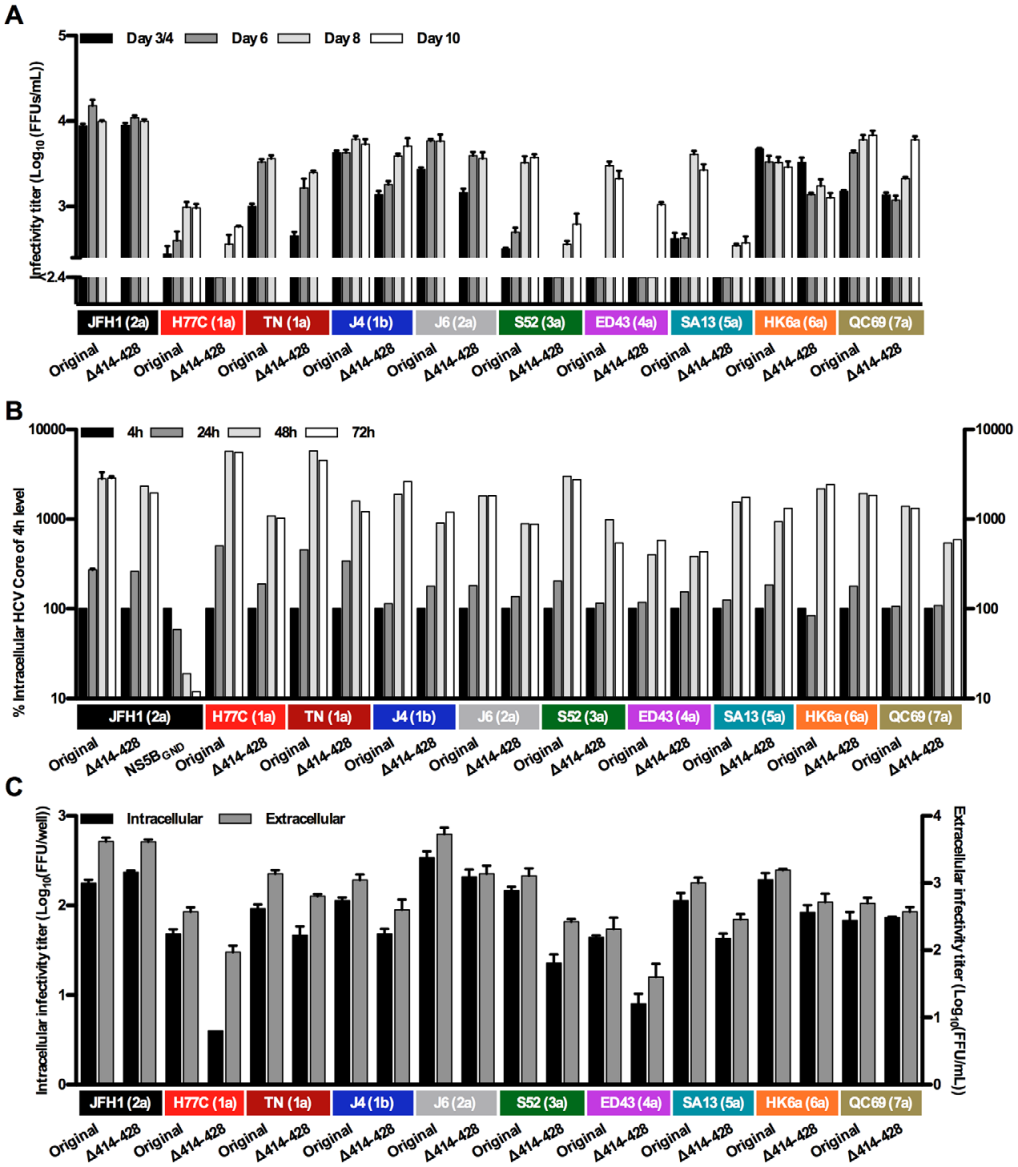
Infectivity titers and replication capacity of HCV NS5A domain III Δ414-428 mutants. (**A**) Infectivity titers in supernatant after transfection of Huh7.5 cells with original NS5A genotype recombinants and Δ414-428 mutants. The lower limit of detection in the experiments shown was 10^2.4^ FFU/mL (indicated by the y-axis break). (**B**) As a measure for HCV RNA replication, S29 cells were transfected for 4 hours and intracellular HCV Core levels were measured after 4, 24, 48 and 72-hours. Values were normalized for transfection efficiency using 4-hour Core amounts of the respective recombinant. The JFH1(2a) NS5B_GND_ mutant was included as a replication negative control. (**C**) Intra- and extracellular infectivity titers 48 hours after transfection of S29 cells. Intracellular titers are given per well of 4×10^5^ transfected S29 cells. Colored boxes indicate the NS5A isolate and (genotype). Error bars indicate SEM of triplicate determinations, except for in (B) where JFH1(2a) was included in duplicates.

In genotype 2a studies, NS5A domain III was reported to have a primary role in virus production, e.g. deletion of JFH1 domain III significantly attenuated virus production while replication was less affected [23], [27], [28]. The high degree of sequence conservation of residues 414-428 (Figure S1) suggested an important function of this region; thus we generated deletion mutants for the NS5A genotype 1–7 recombinants (Figure 1A). While the JFH1(2a) Δ414-428 mutant was not attenuated, infectivity titers after transfection of Huh7.5 cells were decreased up to 10-fold for deletion mutants of other NS5A isolates (Figure 4A). This correlated with previous reports on a 382–428 JFH1 deletion mutant [27]. After spread of infection in culture and passage to naïve cells, sequencing of the ORF from recovered viruses confirmed the deletion for all recombinants and identified changes for the H77C(1a), TN(1a), S52(3a), SA13(5a) and QC69(7a) deletion mutants, primarily in the NS5A region downstream of the deletion and in p7, NS2 and NS3 (Table 1).

**Table 1:** Non-synonymous mutations identified for HCV NS5A Δ414-428 deletion mutants.

		Location of acquired mutations					
Recombinant^A^	Mutant, HCV gene and residues	p7	NS2	NS3	NS4B	NS5A	NS5B
H77C(1a)	NS5A, Δ414-428	F26S (772)	V68A (877)	V389L (1415)	I259T (1970)	D441G (2413) V446M (2418)	
TN(1a)	NS5A, Δ414-428		I64T (873)	A156S (1182) I399T (1425)		L363P (2335)	
S52(3a)	NS5A, Δ414-428		I70T (879)	A380G (1406)		S434P (2406) C447R (2419)	K50T (2470)
SA13(5a)	NS5A, Δ414-428			E79G (1105) H541Y (1567)		S299F (2271) E439K (2411)	
QC69(7a)	NS5A, Δ414-428	F26S (772)					

A Numbering is according to individual H77 reference proteins (AF009606). Numbering according to the H77 reference polyprotein is given in parenthesis. All original cell culture adaptive mutations, as indicated in Materials & Methods, and the engineered deletion were retained. Mutations in the ORF are listed for residues with at least 50% change in the sequence reads. No changes were observed for Δ414-428 mutants of other NS5A isolates.

To analyze whether attenuation of Δ414-428 mutants was caused by reduced replication capacities, we measured intracellular HCV Core after transfection of S29 cells. For most mutants, replication was decreased less than 2-fold, but greater than 3-fold reductions in intracellular Core were observed at multiple time points after transfection with H77C(1a), TN(1a) and S52(3a) mutants (Figure 4B). The effect of deletion mutants on intra- and extra-cellular infectivity after transfection of S29 cells corresponded to observations from Huh7.5 culture supernatant, with greater than 3-fold reductions in titers for the H77C(1a), J6(2a), S52(3a), ED43(4a) and SA13(5a) recombinants; in this assay JFH1(2a) and QC69(7a) were not affected by the deletion (Figure 4C). For most recombinants, reductions in replication capacities could explain the decreases in intra- and extracellular titers observed. However, the less than 2-fold decrease in replication capacity for the ED43(4a) and SA13(5a) mutants is not likely to explain the greater than 3-fold reductions in intra- and extracellular titers observed. This indicated an effect on virus assembly in addition to the effect on replication for certain NS5A isolates.

To determine whether the genotype 2-specific 20 residue insertion immediately downstream of residue 428 (Figure 1A and S1) could be responsible for the efficient replication and virus production of the JFH1(2a) Δ414-428 mutant (Figure 4), we replaced the 414-428 region of the H77C(1a) recombinant by the JFH1-specific insertion. For this mutant, “Δ414-428+20aa”, replication capacity was slightly decreased, but unlike for the H77C(1a) Δ414-428 mutant, no attenuation of intra- or extracellular infectivity was observed and no additional mutations were identified after passage to naïve cells. Thus, the genotype 2-specific insertion could potentially compensate for the deletion of residues 414-428.

**Figure 5:**
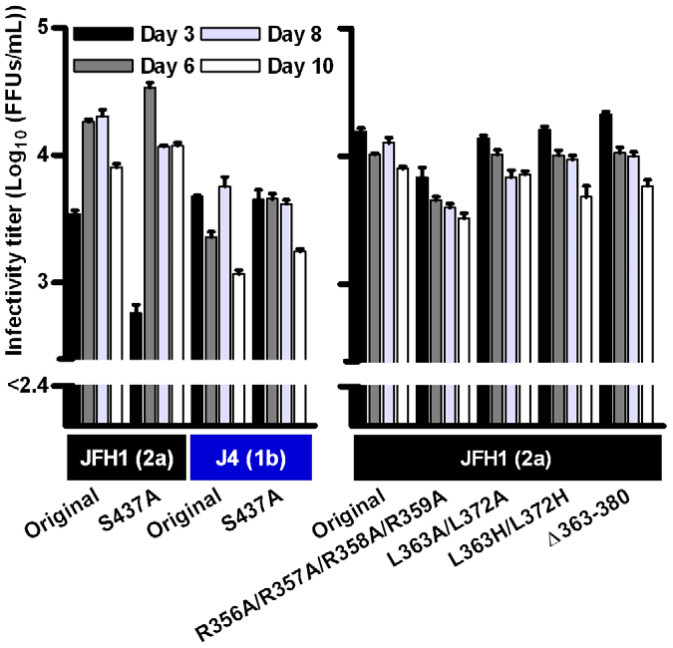
Infectivity titers in supernatant after transfection of Huh7.5 cells with domain III mutants. For the S437A mutants, J4 and JFH1 recombinants are shown as representative examples, since no significant effect on titers was observed for any of the NS5A genotype 1–7 recombinants. For comparison, JFH1(2a) was included in each experiment. The lower limit of detection in the experiments shown was 10^2.4^ FFU/mL (indicated by the y-axis break). Colored boxes indicate the NS5A isolate and (genotype). Error bars indicate SEM of triplicate determinations.

Serines in domain III play an important role for genotype 2a NS5A function [28], [29], [45]; in particular the S437A mutation (Figure 1) was reported to attenuate J6/JFH1 virus production [28]. After transfection of Huh7.5 cells with RNA transcripts of S437A mutants of NS5A genotype 1–7 we did, however, not identify significant attenuation of virus production (Figure 5 and data not shown). No changes were identified in the ORF of the recovered JFH1(2a) S437A mutant and residue 437 had not reverted for mutants of other NS5A isolates.

We further analyzed the conserved arginine/lysine motif at residue 356–359 and the partially conserved 363–380 region that covers the completely conserved residues 363, 372 and 376. Huh7.5 cells were transfected with JFH1(2a) mutants containing point mutations or a deletion of the 363–380 region (Figure 1 and S1). A minor decrease in infectivity titers was observed for the R356A/R357A/R358A/R359A mutant, but not for L363A/L372A, L363H/L372H or Δ363–380 mutants (Figure 5). We confirmed the presence of point mutations and the 363–380 deletion in recovered virus after passage to naïve Huh7.5 cells. Thus, no major effect of the introduced mutations was observed, even after deletion of a larger conserved region of NS5A domain III.

NS5A domain III was previously reported to be important for efficient virus production of genotype 2a [27], [29]. The present findings indicate that domain III is also of importance for replication, with isolate-specific dependence on the highly conserved 414-428 region for replication and virus assembly.

### NS5A stability is decreased by mutations in LCSII and domain III

**Figure 6:**
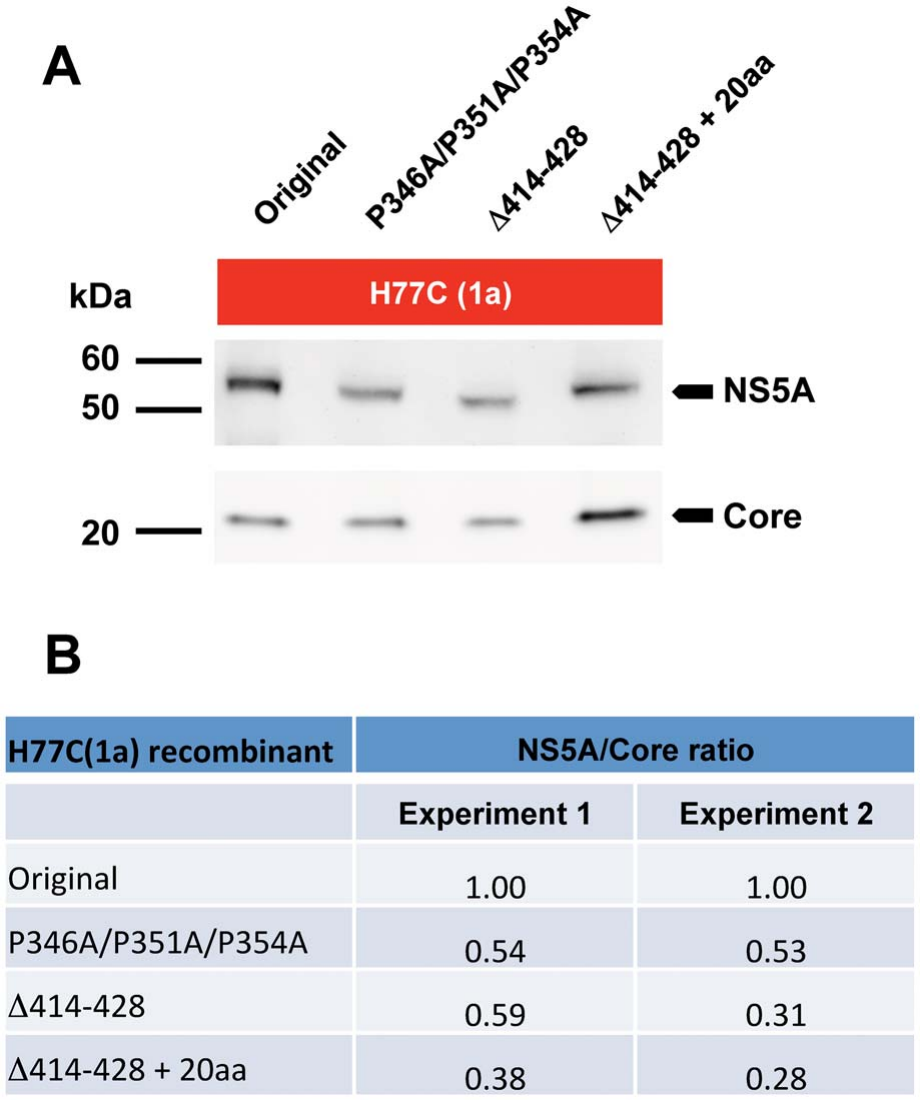
NS5A stability of H77C(1a) mutants. Amounts of NS5A were compared to Core after spread of infection to >80% of Huh7.5 cells transfected with H77C(1a) and P346A/P351A/P354A, Δ414-428 or “Δ414-428+20aa” mutants. (**A**) Western blots from a representative experiment of NS5A (56/58 kDa for original), and Core (21 kDa) detected with antibodies H26 (Abcam) and C7-50 (Enzo Life Science), respectively. (**B**) Ratios between NS5A/Core were calculated after subtraction of background using ImageJ and normalized to the original H77C(1a). Values from two independent transfection experiments are shown.

To further investigate the functional impact of mutations in LCSII and domain III, we analyzed the potential effect on NS5A stability. Due to limitations in available NS5A antibodies recognizing domain III deletion mutants of the different isolates, we focused this analysis on H77C(1a) and the mutants P346A/P351A/P354A in LCSII as well as Δ414-428 and “Δ414-428+20aa” in domain III. Huh7.5 cells were transfected with the various recombinants, and cultures with >80% infected cells where lysed for analysis in western blots. To control for impact on replication, NS5A amounts were normalized to amounts of Core. All analyzed mutants had relative NS5A amounts decreased to 30–50% of that of the original H77C(1a) (Figure 6). Thus, assuming that changes in NS5A did not influence stability of Core, we concluded that the P346A/P351A/P354A mutations in LCSII and the Δ414-428 deletion in domain III decreased stability of H77C(1a) NS5A.

### NS5A changes induced compensatory mutations in p7 and vice versa

As seen in Table 1, two recombinants with modifications in NS5A domain III acquired the F26S (F772S) mutation in p7. We previously reported that this mutation compensated for the exchange of NS5A in the J6(2a) and ED43(4a) recombinants, and in the DH6(1a) NS5A recombinant this residue changed to leucine [12]. To further investigate a putative genetic linkage between NS5A and p7 sequences, we introduced p7 F26S into H77C(1a), TN(1a) and JFH1(2a) NS5A recombinants, which did not rely on adaptation [12]. After transfection and passage to naïve Huh7.5 cells we sequenced p7 and NS5A of viral genomes recovered from supernatants. The H77C(1a) and TN(1a) mutants acquired C447R (C2419R) or V446L (V2418L) in NS5A, respectively, while the JFH1(2a) mutant did not acquire additional mutations (Table 2). This was seen in four separate experiments for each mutant, while four replicate experiments with the original H77C(1a), TN(1a) and JFH1(2a) recombinants did not lead to accumulation of any mutations (Table 2). No variation is observed among genotype 1a and 2a isolates in the HCV database at these two residues. To cross-check whether the observed changes in NS5A of the genotype 1a recombinants compensated for the p7 mutation, we transfected H77C(1a) NS5A_C447R_ and TN(1a) NS5A_V446L_ mutants in triplicates. After passage to naïve cells, all H77C(1a) NS5A_C447R_ cultures and two of three TN(1a) NS5A_V446L_ cultures acquired changes in p7, including F26S (Table 2). Most cultures in addition acquired changes in NS5A. We hypothesized that if the F26S (p7) and C447R or V446L (NS5A) mutations were compensatory, combining these mutations should abolish the need for further mutations. Indeed, after transfection and subsequent passage of H77C(1a) p7_F26S_NS5A_C447R_ and TN(1a) p7_F26S_NS5A_V446L_ mutants in triplicates, no additional mutations were observed except for one additional coding change in NS5A for one TN(1a) p7_F26S_NS5A_V446L_ culture (Table 2). Thus, modifications of NS5A from several isolates induced changes in p7, and p7 mutations induced changes in NS5A for genotype 1a. JFH1(2a) apparently better tolerated the change in p7 and did not rely on compensatory NS5A mutations.

**Table 2:** Non-synonymous mutations in the p7 and NS5A regions of H77C(1a), TN(1a) and JFH1(2a) p7 and NS5A mutants.

		Location of acquired mutations	
Recombinant^A^	Mutation	p7	NS5A
H77C(1a)	None	-	-
	None	-	-
	None	-	-
	None	-	-
	p7_F26S_	-	C447R (2419)
	p7_F26S_	-	C447R (2419)
	p7_F26S_	-	C447R (2419)
	p7_F26S_	-	C447R (2419)
	NS5A_C447R_	*S12I (758)*	*A227V (2199)*
	NS5A_C447R_	Y31C (777), *S43F (789)*	*D126N (2098)*
	NS5A_C447R_	*F19L (765)*, *F26S (772)*	-
	p7_F26S_, NS5A_C447R_	-	-
	p7_F26S_, NS5A_C447R_	-	-
	p7_F26S_, NS5A_C447R_	-	-
TN(1a)	None	-	-
	None	-	-
	None	-	-
	None	-	-
	p7_F26S_	-	V446L (2418)
	p7_F26S_	-	V446L (2418)
	p7_F26S_	-	V446L/M (2418)
	p7_F26S_	-	V446L (2418)
	NS5A_V446L_	-	*D50V (2022)*
	NS5A_V446L_	A28V (774)	*C39G (2011)*, *D427G (2399)*
	NS5A_V446L_	F26S (772) *Y31C (777)*	
	p7_F26S_, NS5A_V446L_	-	I280V (2252)
	p7_F26S_, NS5A_V446L_	-	-
	p7_F26S_, NS5A_V446L_	-	-
JFH1(2a)	None	-	-
	None	-	-
	None	-	-
	None	-	-
	p7_F26S_	S26F (772)	-
	p7_F26S_	-	-
	p7_F26S_	-	-
	p7_F26S_	-	-

A Numbering is according to individual H77 reference proteins (AF009606). Numbering according to the H77 reference polyprotein is given in parenthesis. Only p7 and NS5A were sequenced in these experiments. The engineered mutations were retained, except for one JFH1(2a) p7_F26S_ mutant where the introduced p7 mutation reverted. Mutations are listed for residues with at least 50% change in the sequence reads, except for *italicized* mutations that were minor. A dash indicates that no mutations were identified.

### Functional importance of HCV NS5A genotype-specific residues

**Figure 7:**
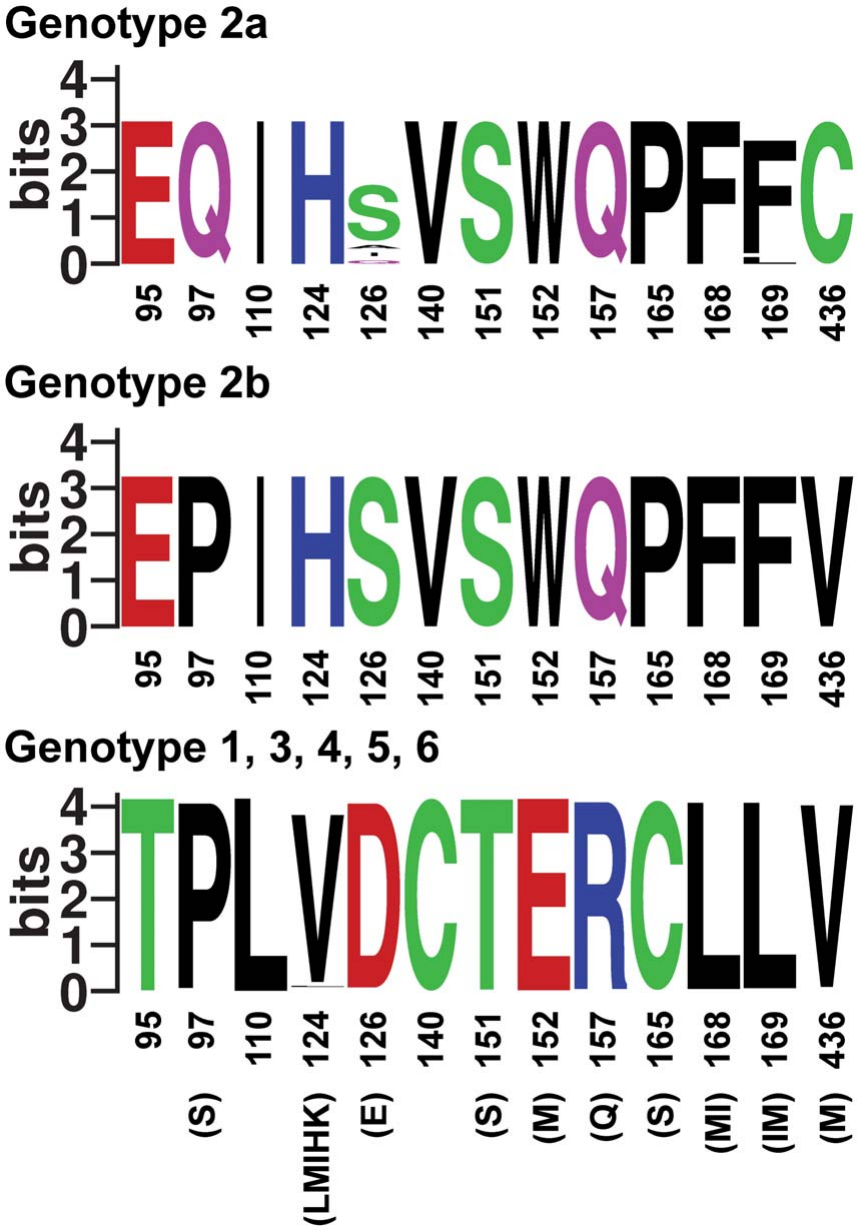
Genotype 2 or 2a-specific positions in HCV NS5A. Residues with genotype 2 or 2a-specificity among isolates in the most recent pre-made NS5A web-alignment consisting of 673 entries from the Los Alamos HCV sequence database, including 12 genotype 2a and 14 genotype 2b sequences, are visualized as a logo plot [63]. The information content at every position is given in bits. Amino acids are colored by properties: polar (green), basic (blue), acidic (red) and hydrophobic (black). JFH1 has isoleucine (I) at position 140. Residues observed at frequencies too low to appear in the sequence logo (but observed in at least two sequences) are given below. Genotype 7a and other subtypes of genotype 2 than 2a and 2b were not included, since no or only one NS5A sequences were available. Numbering is according to H77 reference NS5A sequence AF009606.

**Figure 8:**
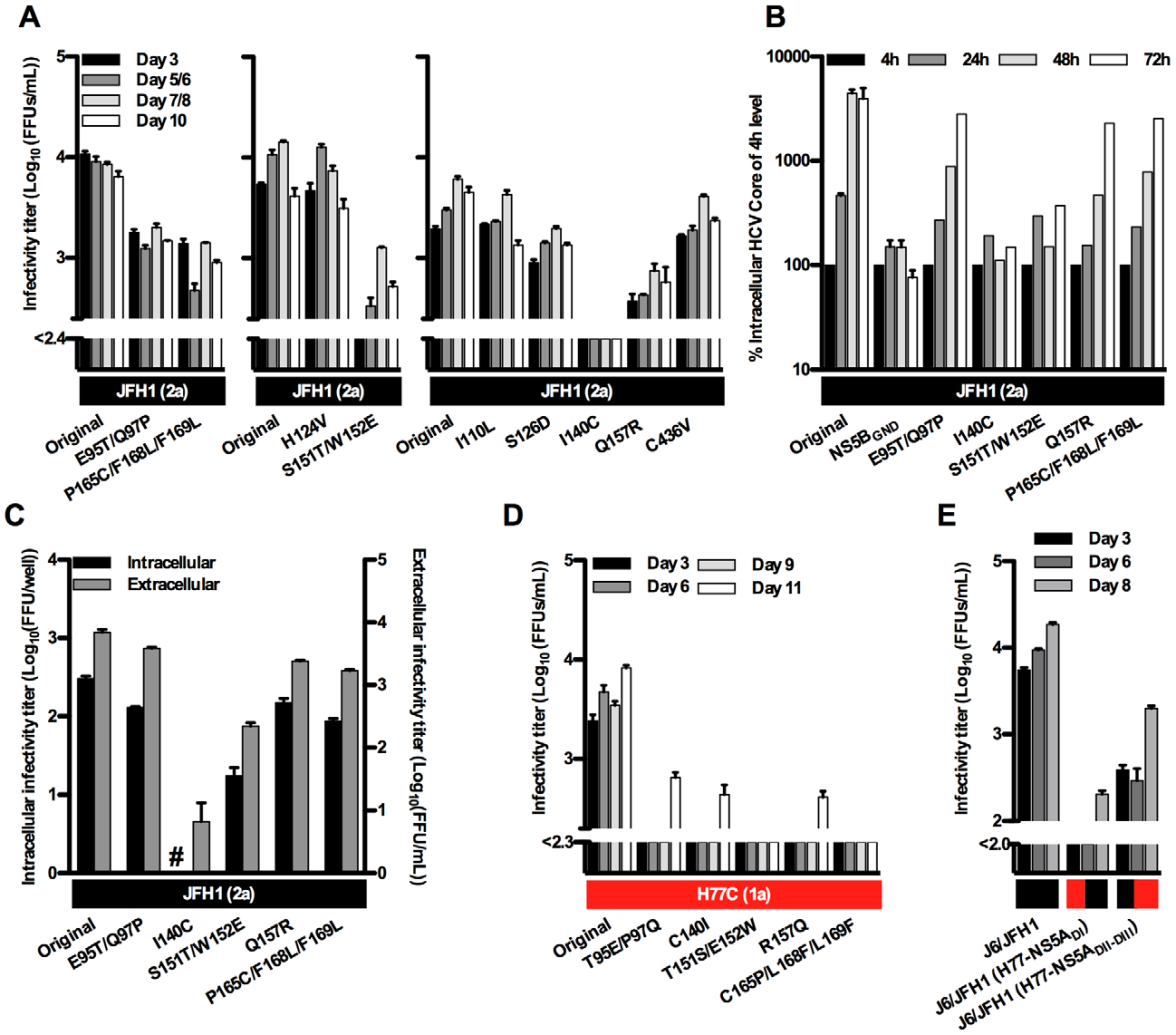
Characteristics of NS5A recombinants with mutations at genotype-specific positions. (**A**) Infectivity was measured in supernatants after transfection of Huh7.5 cells with RNA transcripts from JFH1(2a) with genotype-specific mutations. For comparison, JFH1(2a) was included in each experiment. The lower limit of detection in the experiments shown was 10^2.4^ FFU/mL (indicated by the y-axis break). (**B**) As a measure for HCV RNA replication, S29 cells were transfected for 4, 24, 48 or 72 hours with JFH1(2a) carrying genotype-specific mutations and intracellular HCV Core levels were measured. Values were normalized for transfection efficiency using an average of 4 hour Core amounts from 11 parallel transfections. The JFH1(2a) NS5B_GND_ mutant was included as a replication negative control. (**C**) Intra- and extracellular infectivity titers 48 hours after transfection of S29 cells with JFH1(2a) carrying genotype-specific mutations. Intracellular titers are given per well of 4×10^5^ transfected S29 cells. (**D**) Infectivity titers in supernatant after transfection of Huh7.5 cells with RNA transcripts from H77C(1a) NS5A recombinants with mutations at genotype-specific positions. The lower limit of detection in the experiments shown was 10^2.3^ FFU/mL (indicated by the y-axis break). (**E**) Growth characteristics of domain I exchange recombinants. Huh7.5 cells were transfected with J6/JFH1, J6/JFH1(H77C-NS5A_DI_) or J6/JFH1(H77C-NS5A_DII–III_) and cultures were followed by HCV infectivity titers released to the supernatant. The lower limit of detection in the experiments shown was 10^2.0^ FFU/mL (indicated by the y-axis break). Colored boxes indicate the NS5A isolate and (genotype). #: no FFU were detected. Error bars indicate SEM of triplicate determinations, except for in (B) where data was included from four and three independent experiments with JFH1(2a) and the NS5B_GND_ mutant, respectively.

In phylogenetic analysis of NS5A, genotype 2 clusters separately from other genotypes. To investigate the functional significance of genotype-specific residues in the highly variable NS5A protein, we changed positions in JFH1(2a), where genotype 2a residues were different from all or almost all isolates of genotypes 1, 3, 4, 5 and 6 (Figure 7). Most of these positions were in NS5A domain I, and in most cases genotype 2b isolates had residues identical to genotype 2a. Mutations were introduced into JFH1(2a) singly or combined: E95T/Q97P, I110L, H124V, S126D, I140C, S151T/W152E, Q157R, P165C/F168L/F169L and C436V. After transfection, <10% of Huh7.5 cells were HCV positive for the I140C and S151T/W152E mutants, while around 30% were positive for JFH1(2a) and other mutants. Virus production was attenuated for these two mutants and for the E95T/Q97P, Q157R and P165C/F168L/F169L mutants (Figure 8A). After immediate or delayed spread of infection in the transfection culture, supernatants were passaged to naïve cells and the NS5A gene of recovered mutants was sequenced; the I140C and S151T/W152E mutants both acquired V130I. In addition the I140C mutant acquired L188F, and the S151T/W152E mutant acquired T122M. In two independent experiments, the H124V mutant acquired I289T or S300P in domain II. No mutations in NS5A were observed for the other mutants. In functional analyses we investigated the five mutants attenuated after transfection of Huh7.5 cells. As demonstrated by measurement of intracellular HCV Core after transfection of S29 cells, replication was highly attenuated for the I140C and S151T/W152E mutants while Core accumulation was delayed for the E95T/Q97P, Q157 and P165/F168/F169 mutants (Figure 8B). Intra- and extracellular infectivity titers after transfection of S29 cells were reduced up to 10-fold for the E95/Q97, Q157R, S151T/W152E and P165C/F168L/F169L mutants, while reductions of 100-fold or more were observed for the I140C mutant (Figure 8C). Thus the genotype 2 or 2a-specific residues E95T/Q97P, Q157R and P165C/F168L/F169L seemed to be important both for viral replication and intracellular virus assembly.

To address whether the observed attenuation of JFH1(2a) mutants was indeed due to genotype-specific requirements at the analyzed positions, we introduced the reverse mutations into the H77C(1a) NS5A recombinant. After transfection of Huh7.5 cultures, around 10% HCV positive cells were observed for the T95E/P97Q, C140I, T151S/E152W and R157Q mutants, while 30% were positive for the original H77C(1a) NS5A recombinant; no positive cells were observed for the C165P/L168F/L169F mutant. This was reflected by decreased infectivity titers for all mutants (Figure 8D). Thus, mutation of the H77(1a) NS5A recombinant to genotype 2- or 2a-specific residues also led to attenuation.

Since NS5A function depended on several genotype-specific residues in domain I, we wanted to determine whether this domain could function as a genotype-specific entity. We thus replaced NS5A domain I for JFH(2a) by H77C sequence and for H77C(1a) by JFH1 sequence, thereby generating two J6/JFH1 recombinants with either domain I or domain II–III from H77C. After transfection into Huh7.5 cells, the H77C domain II–III recombinant had slightly delayed viral spread and infectivity titers decreased by more than 10-fold compared to JFH1(2a), while the domain I recombinant was highly attenuated (Figure 8E). Data from our previous study showed attenuation even for a J6/JFH1-based recombinant with domain I from the J6 isolate also of genotype 2a [12]. Thus, NS5A function depended on genotype-specific residues in domain I and genotype-specific interactions existed between NS5A domain I and domain II–III.

## Discussion

Due to the clinical and biological importance, there has been great interest in the study of HCV genotype-specific functional differences [9]. However, most functional studies of HCV in infectious culture systems have depended on a single HCV isolate (JFH1). The NS5A protein was so far primarily studied in genotype 1 and 2 replicon systems or in the JFH1 genotype 2a cell culture system. Interestingly, replicon-enhancing mutations were not permissible in vivo [32], emphasizing that conclusions from the replicon systems should be drawn with caution. In this study, we used infectious NS5A genotype 1–7 recombinants [12], and demonstrated a universal dependence of viral replication on the NS5A amphipathic alpha-helix, domain I, LCSI and domain II. Thus, it was demonstrated that all HCV genotypes require these domains for replication. Interestingly, isolate-specific effects of mutations in LCSII and of deletions in domain II and III revealed novel functional differences between NS5A isolates. Furthermore, NS5A function was shown to depend on genotype-specific residues in domain I, a finding that could influence the effect of directly acting antiviral compounds directed against this NS5A domain. Thus, functional isolate-specific differences are emerging for HCV [3], [42], which will be of critical importance for our understanding of HCV biology and for development of antiviral strategies that target NS5A and other regions with isolate variability.

We addressed the importance of the various regions of NS5A for replication and virus production, by analyzing more than 80 mutants in culture. We showed that viral replication in context of the complete life-cycle was critically dependent on the NS5A amphipathic-alpha helix, domain I, LCSI and domain II for isolates of genotypes 1a, 1b, 2a, 3a, 4a, 5a, 6a and 7a. The absence of HCV positive cells by immunostaining early after transfection corresponded to observations for knockout mutants of non-structural proteins NS3, NS4A, NS4B, and NS5B, and a mutant with an introduced stop-codon, all expected to abolish replication. We demonstrated that reversion could occur even for such single-residue mutants, potentially due to the high error-rate of the T7 polymerase used for RNA in vitro transcription. Thus, abrogation of replication due to mutations shown to disrupt the hydrophobic face of the amphipathic alpha-helix, mutation of two zinc-binding cysteines in domain I, or due to exchange of a conserved tryptophan in domain II confirmed and extended previous findings in the Con-1(1b) replicon system [16], [22], [23]. Contrarily, dependence on LCSI in the infectious cell culture system was demonstrated by the highly attenuated phenotype caused by the replicon-enhancing mutation S225P [33], which was also not permissible in vivo [32]. Thus, at least for this mutant the infectious NS5A cell culture systems reflected findings in vivo better than replicon systems. Recently, S225P was shown to enhance replication but inhibit HCV Core release of the full-length Con-1(1b) isolate in vitro [46], which was not in agreement with our data from infectious culture systems. In theory, inhibition of virus production but not replication could be specific for the Con-1(1b) isolate, however, the Con-1(1b) and J4(1b) NS5A protein sequences deviate by less than 5%. Replication seemed to depend also on highly conserved residues downstream in LCSI (Figure S1), as deletion of J4(1b) NS5A residues 235–282 (Δ47) in this study abolished replication, while residues 246–308 previously were deleted in J6/JFH1 with no apparent effect on viability [27]. Interestingly, we observed decreased infectivity titers and mutations in the C-terminus of NS5A for domain II Δ250–293 mutants of genotype 1a but not 2a (Figure 2 and [39]). Thus, HCV apparently shows an isolate-specific dependence on the highly variable N-terminal region of NS5A domain II. The genotype 2 protein sequence deviates in this region from most other genotypes (Figure S1), potentially reflected by differences in structure or function and thereby also in the effect of the deletion. With the critical dependence on selected residues or regions of the NS5A amphipathic alpha-helix, domain I, LCSI and domain II for replication, these regions would be obvious targets for antiviral therapy [47].

Great differences in the effect on viral infectivity titers were observed among the NS5A genotype 1–7 mutants when residues 346, 351 and 354 in LCSII were mutated, with J4(1b) and JFH(2a) being least affected. Measurements of intracellular HCV Core showed that most NS5A LCSII mutants had up to 10-fold reduced replication capacities, while ED43(4a) and QC69(7a) mutants were severely attenuated. Greater than 10-fold reductions in intra- and extracellular infectivity for the H77C(1a), S52(3a), SA13(5a) and HK6a(6a) mutants indicated an additional effect on virus production for these isolates. Findings on the JFH1(2a) LCSII mutant were in agreement with previous findings that a Con-1(1b) but not a JFH1(2a) replicon depended on P346 for replication, while mutation of the infectious full-length JFH1 recombinant led to lower levels of infectivity [42]. In line with this previous study, we found that mutation of P346/P351/P354 inhibited replication at an isolate-specific level. Interestingly, replication of the J4(1b) mutant was only slightly decreased, while the Con-1(1b) P346A replicon mutant was severely attenuated [42]. This might be due to differences between the isolates or between the two in vitro systems studied. Our QC69(7a) LCSII mutant lacked 14 nucleotides in the poly-pyrimidine tract (Materials & Methods), however, it is unlikely that this caused the observed attenuation of replication since previous studies demonstrated efficient replication of J6/JFH1 with much shorter poly-pyrimidine tracts [48]. In agreement with previous findings for residue 343 mutants [42], we could not confirm dependence on this position [23] for replication of any of the NS5A recombinants.

Deletion of the highly conserved residues 414-428 led to isolate-specific decrease in infectivity titers and replication (Figure 4). The deletion had very limited effect on the JFH1(2a) mutant, while most other NS5A isolate mutants had reduced replication capacities; most pronounced decreases were observed for H77C(1a), TN(1a) and S52(3a) mutants. Reductions in replication capacities might explain the decrease in intra- and extracellular titers observed for most mutants, however, the less than 2-fold reduction in replication capacity for ED43(4a) and SA13(5a) mutants compared to the greater than 3-fold reduction in produced infectious particles indicated an effect of the 414-428 deletion on assembly of intracellular infectious particles for these recombinants. The finding that a genotype 1a mutant with residues 414-428 replaced by the downstream genotype 2-specific insertion of 20 residues produced infectivity titers comparable to the original NS5A recombinant, indicated that this insertion could compensate for the 414-428 deletion. Others previously studied JFH1 mutants without the 382–428 region [27] or the genotype 2 insertion sequence [49] and found only minor effects on replication, production of infectious particles and co-localization of NS5A and Core on lipid droplets [27]. This correlated with our results with the JFH1(2a) 414-428 deletion mutant. However, deletion of the 408–437 region [29], which covered the 414-428 region and the genotype 2 -specific sequence, or the almost entire domain III (residues 356–439) [27] of genotype 2a, was highly attenuating for viral infectivity but not for replication. Thus, domain III residues outside the 414-428 region appear to be the most important determinants of efficient virus production.

Experiments with H77C(1a) demonstrated that selected LCSII and domain III mutants led to decreased amounts of NS5A normalized to Core protein levels. Assuming that Core stability was not affected by introduced mutations, this suggested a decreased stability of the NS5A protein. Similar observations were recently reported when two or four of the serines S432, S434, S437 and S438 were changed to alanine in a JFH1 recombinant carrying NS5A domain III from H77 [50]. Such effects could possibly be associated with disruption of NS5A co-localization with Core on lipid droplets [27] or disruption of interactions with annexin A2 [51].

The J6/JFH1 S437A mutation was previously shown to significantly decrease infectivity titers 48 hours after transfection [28], however, another study indicated that mutation of at least two of the S432, S434 and S437 residues was required for a significant reduction of particle production and impaired NS5A-Core interaction, and that viral kinetics were attenuated only early after transfection [29]. Similar findings were reported in the JFH1 background with H77 NS5A domain III [50]. This might explain why we observed only a less than 10-fold reduction in infectivity titers on day 3 but not thereafter (Figure 5). Surprisingly, deletion or point mutation of the relatively conserved 363–380 region in domain III (Figure S1) did not significantly affect JFH1(2a) virus production. Similarly, mutation of arginines 356–359 in a putative nuclear localization signal [52] led to only slight reduction of infectivity titers for JFH1(2a). Most interestingly, changing highly conserved residues in NS5A LCSII and domain III of various HCV isolates led to very different effects on replication and virus production. This emphasizes the evolutionary development of functional differences among the HCV genotypes in particular in variable regions of the genome [3]. Investigation of the role of these regions in vivo would be of interest for future studies.

When replacing the entire NS5A gene of J6/JFH1 with that of other isolates [12], or conducting NS5A reverse genetic experiments as done here, putative compensatory mutations were observed outside of NS5A; particularly in p7, NS2 and NS3 (Table 1, [12]). The F26S mutation in p7 provided adaptation to several NS5A recombinants [12], and in this study F26S was acquired by NS5A Δ414-428 deletion mutants. In addition, the p7 mutation also adapted J6/JFH1 recombinants with genotype-specific NS3/4A protease [53]. Interestingly, introduction of F26S into the genetically stable H77C(1a) and TN(1a) NS5A recombinants led to compensatory mutations in NS5A domain III. Moreover, introduction of these NS5A mutations led to mutations in p7, while combining the p7 and NS5A mutations led to stable recombinants. These findings indicated a genetic linkage between NS5A and p7. NS5A interactions might also involve NS3/NS4A, where several Δ414-428 mutants acquired changes, possibly in concert with p7 or other proteins in the Core-NS2 region, since I399T in NS3 (I1425T) acquired by the TN(1a) mutant also adapted a Core-NS2 genotype 1a recombinant [54]. Additionally, a number of potentially compensatory mutations for NS5A deletion mutants in the third transmembrane domain of NS2 (Table 1), for the J4(1b) NS5A recombinant [12], and for Core-NS2 recombinants [7], indicated an interaction between NS5A and NS2. Pull-down, co-localization and reverse genetic experiments demonstrated NS2 interactions with E1, E2, p7, NS3 and NS5A, and studies of compensatory mutations identified the importance of such interactions during production of virus particles [55]–[58]. Furthermore, the p7 F26L mutation was shown to compensate for changes in Core [59], possibly reflecting involvement of these proteins in viral assembly. Unfortunately, useful antibodies targeting p7 are scarce and the protein can not be directly detected in immunostainings. Tagging of p7 was not compatible with efficient virus production (data not shown and [56]), and was therefore not a useful approach for studying protein interactions likely to take place during viral assembly and release. Thus, despite elaborate efforts we failed to establish co-immunoprecipitation or co-localization based evidence for an interaction between p7 and NS5A in the infectious culture system; better reagents are needed for conclusive studies on p7 interactions.

Mutation from genotype 2a to 1a or vice versa of the NS5A genotype 2 or 2a-specific residues 95/97, 140, 151/152, 157 or 165/168/169 led to attenuation in cell culture. This was of particular interest, since the introduced amino acids led to attenuation irrespective of the presence of these amino acid residues at the given position in numerous infectious HCV isolates. This indicated that genotype 2 evolved specific sequence requirements for function of NS5A domain I. Putative genotype-specific compensatory mutations were identified for the JFH1(2a) H124V mutant that changed serine at position 300 (domain II), present in all genotype 2 and only few other isolates, and the JFH1(2a) S151T/W152E mutant that changed threonine at position 122, present only for genotype 2 and 1b isolates. Residues 130 and 188 that changed in the I140C culture are located in close proximity to residue 140 in the NS5A domain I structure [19], suggesting a functional interaction between these residues. Compensatory mutations in domain II and the reduced viability of domain I exchange recombinants suggested important genotype-specific interactions between domain I and other regions of NS5A. Such interactions might in particular be between domain I and II, since a recent study demonstrated that domain III alone could be exchanged between H77 and JFH1 recombinants [50]. To our knowledge this is the first time the function of an HCV protein was found to depend on genotype-specific residues. Since NS5A inhibitors under current development target domain I [14], [47], this may pose challenges for future antiviral therapy.

Although this study significantly increases the number of studied NS5A isolates, only a single isolate was analyzed for most genotypes. Since differences were observed between isolates of the same genotype, e.g. between domain II deletion mutants of H77C(1a) and TN(1a), studies of more isolates will be important to discriminate between isolate- and genotype-specific findings. Since the original NS5A recombinants used in this study all were efficient without requirement for further adaptation, any effects observed for NS5A mutants are likely to be attributed to the particular NS5A isolate. However, it is possible that particular mutations would render NS5A non-functional in the J6/JFH1 genetic background but not in a full-length background of that particular isolate. Thus, it will eventually be of importance to develop full-length cell culture systems for all HCV genotypes.

In conclusion, we demonstrated that all major genotypes depended on the NS5A amphipathic alpha-helix, domain I, LCSI and domain II for viral replication. Interestingly, dependence on LCSII and domain III for HCV RNA replication and virus production varied with the NS5A isolate. Additionally, functional genotype-specific differences of NS5A domain I residues were identified. Our study highlights the emerging evidence of significant functional differences between diverse HCV isolates. Observed differences in NS5A will be important to consider in functional understanding and therapeutic targeting of this protein. Further studies in vitro and in vivo will be important for understanding and targeting this pleiotropic viral protein.

## Materials and Methods

### Construction of HCV recombinants

Reverse genetic studies were done with the J6/JFH1 recombinant [31], and the J6/JFH1-based NS5A genotype 1–7 recombinants H77C(1a), TN(1a), J4(1b)_R867H,C1185S_, J6(2a)_F772S_, S52(3a)_D1975G_, ED43(4a)_F772S,Y1644H,E2267G_, SA13(5a)_R1978G,S2416G_, HK6a(6a)_I2268N_ and QC69(7a), expressing the entire NS5A protein (Numbering of mutations according to the H77 reference polyprotein) [12]. Culture adaptive mutations in NS5A are indicated in Figure S1. All mutations analyzed in this study were introduced using site-directed mutagenesis. Marker mutations (according to the H77 reference ORF sequence) introduced to exclude contamination in studies of reversion were T3431C (NS3_stop_), G3830A (NS3_pro_), C4280T (NS3_hel_), G5369A (NS4A_G21V_), C6224T (NS4B_W252S_), C6419T (NS5A_C57G_), C6440T (NS5A_C59G_), T2719C or C8558T (NS5B_GND_). The complete HCV sequence of final plasmid preparations was confirmed, except for NS5A domain mutants that did not acquire mutations in the ORF after passage in cell culture and did not produce decreased infectivity titers after transfection. Sequencing identified the following exceptions; J6/JFH1_P165C/F168L/F169L_ carried the additional non-coding C5485T mutation. The J4(1b) Δ414-428 and QC69(7a) P346A/P351A/P354A mutants lacked 2 and 14 nucleotides in the poly-pyrimidine tract, respectively.

### Culturing, transfection, infection and evaluation of cell cultures

Culturing of Huh7.5 hepatoma cells [31] was done as described [60]. One day before transfection or infection, 4×10^5^ cells were plated per well in six-well plates. In vitro transcription of RNA was described previously [5]. For transfection, 2.5 µg RNA were incubated with 5 µL Lipofectamine2000 (Invitrogen) in 500 µL Opti-MEM (Invitrogen) for 20 min at room temperature. Cells were incubated with transfection complexes for 16–24 hours in growth medium. The individual transfection efficiencies of 20 independent experiments, as measured by HCV Core ELISA (see below) after 4 hours, varied less than 2-fold from the positive control. Intra- and extracellular infectivity titers after transfection of S29 cells [40] were determined as described [61]. For infection experiments, cells were inoculated with virus-containing supernatant for 16–24 hours. Supernatants collected during experiments were sterile filtered and stored at −80°C.

Infected cultures were monitored by immunostaining using mouse anti-HCV Core protein monoclonal antibody (B2, Anogen) as described [5], [60]. Infectivity titers were determined by adding 100 µL of triplicate sample dilutions (diluted 1∶2 or more) to 6×10^3^ Huh7.5 cells/well plated out the day before on poly-D-lysine-coated 96-well plates (Nunc). Cells were fixed and immunostained for HCV 48 hours after infection using a previously established protocol [60]. Primary antibody was Hepatitis C Virus NS3 antibody (H23, Abcam). The previously used anti-NS5A 9E10 antibody [31] gave no signal for the J6(2a) NS5A recombinant and for Δ414-428 mutants and suboptimal signals for several other NS5A recombinants. The number of focus-forming units (FFU) was determined by manual counting or on an ImmunoSpot Series 5 UV Analyzer (CTL Europe GmbH) with customized software as previously described [61]. HCV RNA quantification was done using an in-house assay as described [60].

### HCV Core ELISA

For measurement of intracellular HCV Core, 10^5^ S29 cells [40] per well plated the day before in 24-well plates were transfected with HCV RNA transcripts for the indicated time period. After 4, 24, 48 and 72 hours, cells were trypsinized, centrifuged at 1000× g for 5 minutes at 4°C, washed in cold PBS and lysed in cold RIPA-buffer supplemented with protease inhibitor cocktail set III (Calbiochem). Cell lysates were clarified at 20,000× g for 15 minutes at 4°C before measuring HCV Core levels using ORTHO HCV antigen ELISA test kit (Ortho Clinical Diagnostics).

### Western blotting

Huh7.5 cells were trypsinized, washed in cold PBS and lyzed in 200 µl RIPA buffer (Thermo Scientific) with protease inhibitor cocktail set III (Calbiochem) on ice for 10 min. Lysates were treated with RQ1 DNase (Promega) to reduce viscosity, and clarified by centrifugation at 20,000×g for 15 min at 4°C. Protein lysates were loaded on 10% Bis-Tris gels (Invitrogen) and subsequently transferred to 0.45 µm Hybond-P PVDF membranes (GE Healthcare Amersham). Following overnight incubation with specific antibodies (anti-core C7-50, Enzo Life Science) or anti-NS5A (H26, Abcam) at 4°C, unsaturated chemiluminescense images were acquired and protein amounts were quantified based on band intensity using ImageJ.

### Sequence determination of culture-derived HCV

RNA extraction, RT-PCR and direct sequence analysis (Macrogen Inc) [60] as well as primers specific for the NS5A region [12] were previously described. Sequence analysis was performed with Sequencher (Gene Codes Corporation). HCV sequences were retrieved from the European HCV database and the Los Alamos HCV sequence database. Sequence logos were done using WebLogo [62].

## Supporting Information

Figure S1 NS5A alignment of the original amino acid sequence of isolates used for NS5A genotype recombinants. Identical residues are shaded in light blue, conservative in grey and blocks of similar in green. Reference numbering is according to the H77 NS5A reference sequence (AF009606); corresponding numbering of individual isolates is given at the beginning of each section. The complete lengths of the individual isolates are: H77C and TN (genotype 1a), 448 residues; J4 (1b), 447; JFH1 and J6 (2a), 466; S52 (3a), 452; ED43 (4a), 445; SA13 (5a), 450; HK6a (6a), 451; and QC69 (7a), 446. Residues with isolate specific cell culture adaptive mutations are highlighted in red boxes (see Materials & methods); corresponding numbering according to the polyprotein is given in parentheses for consistency. Black boxes highlight residues analyzed in the present study and the NS5A isolate in which mutations were introduced. Regions deleted in reverse genetic studies are indicated with red horizontal lines below the alignment of the given residues. The genotype 2 –specific insertion from JFH1 inserted into the H77C(1a) Δ414-428 mutant was ESDQVELQPPPQGGGVAPGS.
(TIF)

Figure S2 Comparison of intracellular HCV RNA and Core levels. Huh7.5 cells were infected with three different doses of J6/JFH1 (MOI = 1, 0.1 or 0.01) and intracellular levels of HCV RNA (**A**) and Core (**B**) were quantified after 4, 24, 48 and 72 hrs. (**C**) 48 hour (closed symbols) and 72 hour (open symbols) levels of HCV Core (log_10_[fmol/L]) are plotted against HCV RNA (log_10_[IU/mL]) to illustrate the linear relationship between the two measures of HCV replication. The lower limit of detection in the experiments shown was 1450 fmol/L HCV Core (indicated by the y-axis break).
(TIFF)
