# Supplementary figures and images for: Analysis of Functional Differences between Hepatitis C Virus NS5A of Genotypes 1–7 in Infectious Cell Culture Systems
Source: PLoS Pathog. 2012 May 24;8(5):e1002696. doi: 10.1371/journal.ppat.1002696 (PMC3359982; doi:10.1371/journal.ppat.1002696)

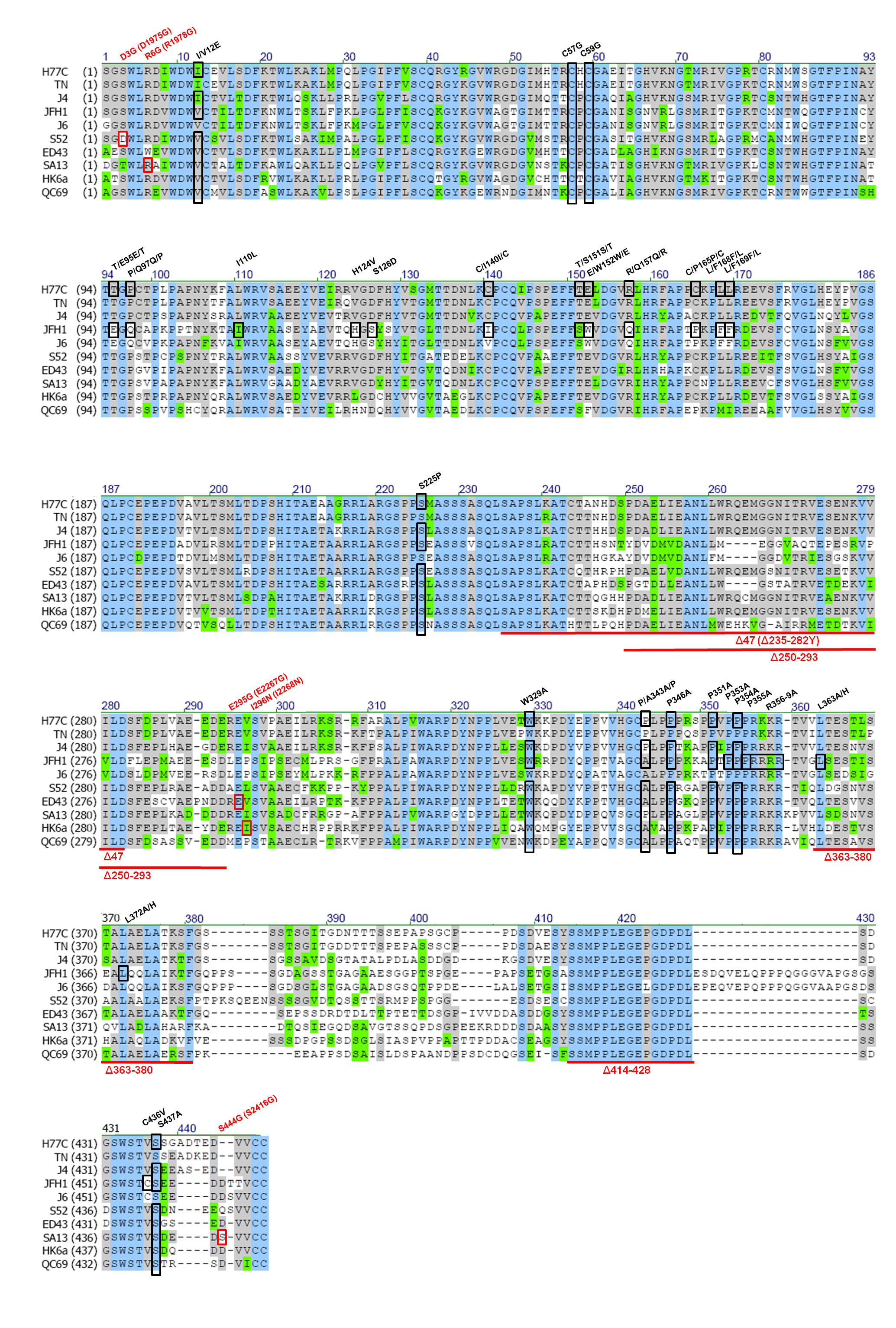

Supplement: Figure S1 — NS5A alignment of the original amino acid sequence of isolates used for NS5A genotype recombinants. Identical residues are shaded in light blue, conservative in grey and blocks of similar in green. Reference numbering is according to the H77 NS5A reference sequence (AF009606); corresponding numbering of individual isolates is given at the beginning of each section. The complete lengths of the individual isolates are: H77C and TN (genotype 1a), 448 residues; J4 (1b), 447; JFH1 and J6 (2a), 466; S52 (3a), 452; ED43 (4a), 445; SA13 (5a), 450; HK6a (6a), 451; and QC69 (7a), 446. Residues with isolate specific cell culture adaptive mutations are highlighted in red boxes (see Materials & methods); corresponding numbering according to the polyprotein is given in parentheses for consistency. Black boxes highlight residues analyzed in the present study and the NS5A isolate in which mutations were introduced. Regions deleted in reverse genetic studies are indicated with red horizontal lines below the alignment of the given residues. The genotype 2 –specific insertion from JFH1 inserted into the H77C(1a) Δ414-428 mutant was ESDQVELQPPPQGGGVAPGS. (TIF) [file ppat.1002696.s001.tif]

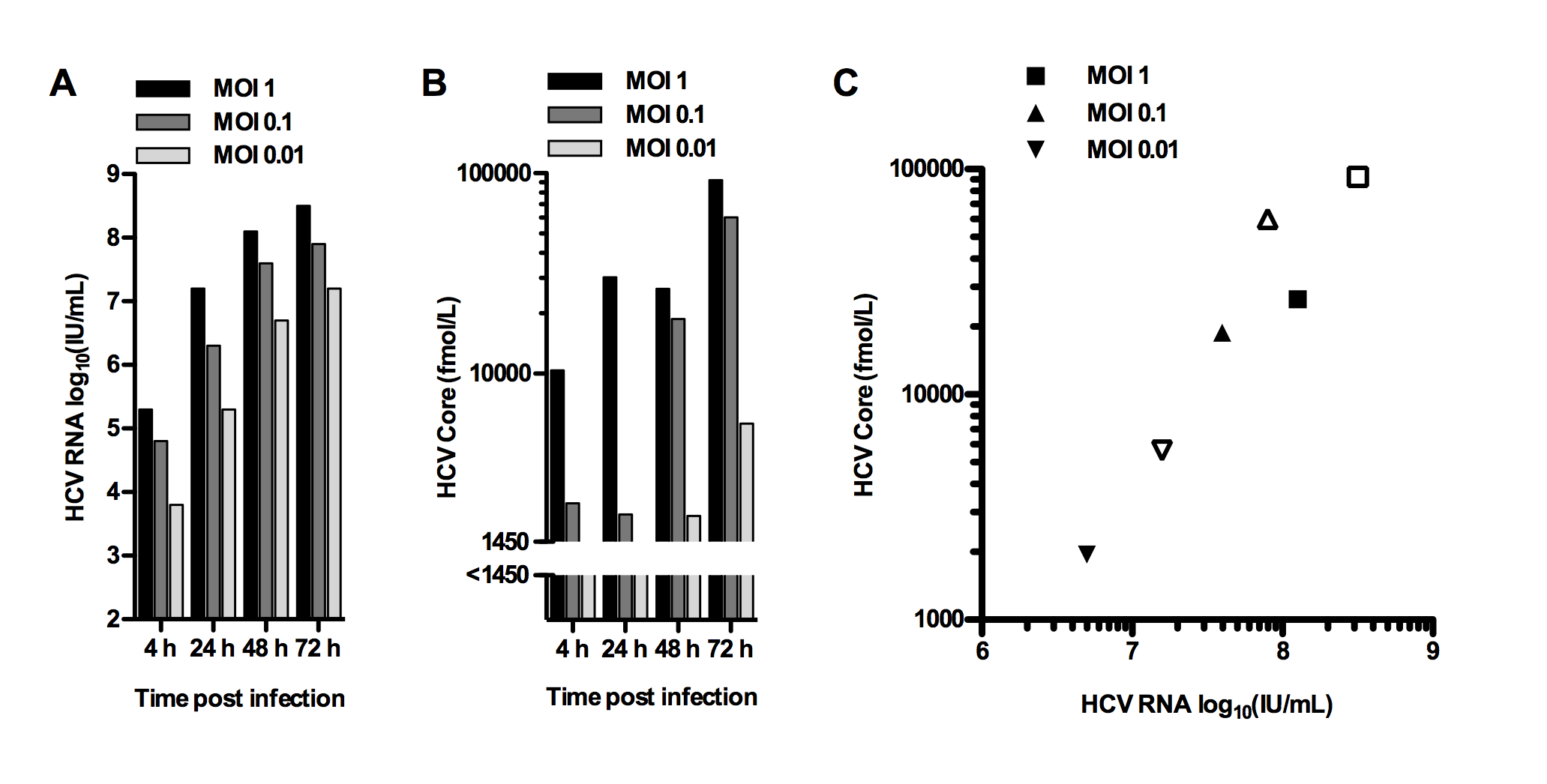

Supplement: Figure S2 — Comparison of intracellular HCV RNA and Core levels. Huh7.5 cells were infected with three different doses of J6/JFH1 (MOI = 1, 0.1 or 0.01) and intracellular levels of HCV RNA (A) and Core (B) were quantified after 4, 24, 48 and 72 hrs. (C) 48 hour (closed symbols) and 72 hour (open symbols) levels of HCV Core (log10[fmol/L]) are plotted against HCV RNA (log10[IU/mL]) to illustrate the linear relationship between the two measures of HCV replication. The lower limit of detection in the experiments shown was 1450 fmol/L HCV Core (indicated by the y-axis break). (TIFF) [file ppat.1002696.s002.tiff]
